# Spanish Physical Education Teachers’ Perceptions about Their Preparation for Inclusive Education

**DOI:** 10.3390/children9010108

**Published:** 2022-01-14

**Authors:** Jorge Rojo-Ramos, Fernando Manzano-Redondo, José Carmelo Adsuar, Ángel Acevedo-Duque, Santiago Gomez-Paniagua, Sabina Barrios-Fernandez

**Affiliations:** 1 Social Impact and Innovation in Health (InHEALTH) Research Group, Faculty of Sport Sciences, University of Extremadura, 10003 Cáceres, Spain; 2 Promoting a Healthy Society Research Group (PHeSO), Faculty of Sport Sciences, University of Extremadura, 10003 Cáceres, Spain; fmanzanoa@alumnos.unex.es (F.M.-R.); jadssal@unex.es (J.C.A.); 3 Facultad de Administración y Negocios, Observatorio de Políticas Públicas, Universidad Autónoma de Chile, Providencia 7500912, Chile; angel.acevedo@uautonoma.cl; 4 BioẼrgon Research Group, University of Extremadura, 10003 Cáceres, Spain; sgomezpa@alumnos.unex.es

**Keywords:** inclusive education, teacher preparation, physical education, perceptions, attitudes, self-perception, special needs, inclusive education

## Abstract

The prevailing rights and quality of life approaches call for the inclusion of people with diversity and/or disabilities in society, including their participation in the educational system. Therefore, different institutions are urging countries to take action to ensure that students with disabilities receive the accommodations and supports they need within the framework of inclusive education. The idiosyncrasies of physical education (PE) classes can be an opportunity to encourage the participation and inclusion of these students. Thus, this study aims to evaluate the PE teachers’ perception about their preparation to address inclusive education. The study involved 260 Spanish primary and secondary PE teachers who answered a sociodemographic questionnaire, three dichotomic questions about their initial and ongoing preparation and the Evaluation of Teacher Training for Inclusion Questionnaire (CEFI-R). PE teachers believe that they have not received the necessary initial preparation and they consider it important to assist in ongoing courses to address their students’ diverse needs. PE teachers are aware of the importance of inclusive education and perceive greater difficulties in secondary education. PE teachers also showed a good predisposition to teach students with special educational support needs, especially found in primary school teachers through the CEFI-R Dimension 1, with statistically significant differences.

## 1. Introduction

Inclusive education is defined as the process of identifying and responding to the diversity of learners’ needs, seeking increased learning and participation in their communities and reducing social exclusion [1]. Although there is awareness about how the educational system should look in the 21st century, in some cases the educational systems are not following the required transformation, failing to provide the learning and participation opportunities to which every student is entitled [2]. This is a challenge, as nowadays the heterogeneity of students is the norm. For this reason, different institutions call for action so that education systems promote attention to diversity, both for students with disabilities and other types of diversity (sexual, ethnic, cultural) in offering quality and inclusive education, without segregation and ensuring that all the students have access to the reasonable adjustments they need [3,4,5]. In this sense, The United Nations (UN) and The United Nations Educational, Scientific and Cultural Organization (UNESCO) highlight the necessity to ensure inclusion and equitable quality education and promote lifelong learning opportunities for every human being [4,6] through a preparation based on the principles of inclusion and equity due to the necessity for an inclusive curriculum which can respond to the students’ diversity in different contexts and settings [7]. The Incheon Declaration Framework [4] proposes actions for the implementation of Sustainable Development Goal 4, Quality Education [6], to ensure inclusive and equitable quality education and promote lifelong learning opportunities for each individual. In addition, Salamanca’s Declaration states that “regular schools should accommodate all children, and those children have the right to be educated together, regardless of their physical, intellectual, social, emotional and linguistic conditions” (p. 59) [3]. In Spain, the current legislation is the 8/2013 Organic Law for the Improvement of Educational Quality (LOMLOE) [8], which regulates the education system. This law emphasizes the value of educational inclusion following the principles of the Convention on the Rights of Persons with Disabilities [9]. In addition, each Spanish region must develop its own educational regulations. In the case of the Extremadura region, the 228/2014 Decree regulates the educational response to diversity, helping stakeholders to make decisions in the provision of human and material supports and adaptations [10]. It is worth noting that in Spain (2018), there were 219,720 students (32.9%) with special educational needs derived from disability, while in Extremadura they represented 1.6% [11].

However, a set of barriers must be overcome to achieve an inclusive school, including inflexible teaching systems focused on conceptual content, the lack of shared responsibility among educational agents or the lack of leadership, which can cause frustration among teachers [12,13]. For this purpose, guidelines such as those by Booth and Ainscow’s (2011) are recommended to guide the actions to follow along three dimensions: (1) creating inclusive cultures (building community and establishing inclusive values); (2) establishing inclusive policies (creating a school for all and organizing support for diversity); and (3) developing inclusive practices (building a curriculum for all and orchestrating learning) [14].

Teachers have a great responsibility in achieving inclusive education, as they must organize the educational response, design and develop personalized educational practices, motivate students, work collaboratively and use different resources and technologies [15,16,17]. In this sense, teachers’ competencies should include: (1) valuing diversity, (2) supporting and having high expectations of students, (3) working in a team, and (4) developing their professional and personal dimension [18]. Two of the main factors for teachers to succeed in inclusion are related to their preparation, including initial and ongoing [19], and direct contact with students with educational needs during their preparation [20]. In line with this, The European Agency for Development in Special Needs Education [21] proposes that, to acquire these competencies, the initial training of future teachers should include first-hand work experience with students with different needs and teachers with experience in inclusive environments [22]. This leads to the concept of attitude, defined as a position or orientation of thought, which translates into a particular way of thinking, acting or reacting, and is made up of three dimensions: cognitive (beliefs), affective (feelings) and behavioral (actions) [23]. When translated into inclusive education it means “the set of perceptions, beliefs, feelings for or against, and ways of reacting to the educational stance that focuses on the achievement of learning for all students” (p. 53) [20]. Some studies warn us that sometimes teachers are not responsive enough to diversity [24]. Another relevant factor is self-efficacy which refers to “a teacher’s belief in his/her ability to successfully cope with tasks, obligations and challenges related to his/her professional role” (p. 2) [25], considered one of the most important moderators of attitudes [26]. In this way, teachers with high levels of self-efficacy report higher levels of job satisfaction, while lower levels are related to job stress and difficulties in dealing with students’ misbehaviors [27]. Sex doesn’t seem to affect attitudes, although some studies reported better attitudes in women [28]; regarding sex differences in self-efficacy, more research is needed [29]. Although years of experience are important, they are not a guarantee of positive attitudes towards inclusion [20,30]. Therefore, a profound reflection is needed on university curricula and the approach used to prepare future teachers to achieve these competencies [31], which should not only include theoretical knowledge and methodological tools but attitudes towards diversity, professional beliefs and self-efficacy, which should also be considered [32,33,34,35,36,37].

The inclusion of students with disabilities in PE classes without neglecting the rest of the students has been a challenge for PE teachers [38]. Therefore, when referring to disability in the school setting, not only the students with physical, sensory or cognitive impairments must be considered, but also the social context in which he/she must interact [39]. Students with disabilities are committed to inclusion in PE lessons, as they find the opportunity to benefit from a greater number and diversity of activities to share with their non-disabled peers [40]. As mentioned, PE teachers’ attitudes and self-efficacy are considered essential for the inclusion process, favoring or obstructing learning and participation during the PE classes, depending on whether they are positive or negative [41,42]. However, PE teachers often do not feel prepared or self-confident enough to address this task [28]. On the one hand, some PE teachers understand that students with and without disabilities will gain social and learning benefits during PE lessons [43]. On the other hand, others believe that the inclusion of students with high support needs is either problematic or impossible [44] highlighting their low self-efficacy feelings to face their needs [45]. Some PE teachers claim that these students should not attend regular PE classes, reflecting inadequate preparation or the lack of resources and means [46]. Among the PE teachers’ concerns are that inclusive PE classes are more difficult to plan and implement, the difficulties in managing behavioral problems [46] or the lack of adequate facilities and resources [47].

Therefore, this study aims to analyze the perception of Spanish PE teachers concerning their preparation to cope with the variety of educational needs that their students may present and towards inclusive education. This update of scientific knowledge subsequently will allow the proposal of measures to support educative inclusion in general, and specifically in PE lessons.

## 2. Materials and Methods

### 2.1. Participants

The sample was made up of 260 primary and secondary PE teachers from public schools in Spain. The mean of the years of teaching experience was 13.4 years (standard deviation 9.5). A non-probabilistic sampling method based on convenience sampling was used [48].

### 2.2. Procedure

A socio-demographic questionnaire, three dichotomous questions and a tool to measure perceptions about their preparation for inclusion were administered using the Google Forms tool, since e-questionnaires allow savings in costs and time, obtaining a higher rate of return [49]. The time to complete their participation in the study was estimated at 10–15 min. Data were stored directly in a spreadsheet.

The study took place between September and December 2020. To access the sample, an e-mail was sent to all public school PE teachers in public primary and secondary schools in the region of Extremadura (Spain), providing information about the aim of the study, an informed consent form and the URL to fulfil the instruments. The participation was voluntary, after providing informed consent to the research team.

All data were collected anonymously and kept private. The study was conducted according to the guidelines of the Declaration of Helsinki and approved by the Bioethics and Biosafety Committee at the University of Extremadura (protocol code: 186/2021).

### 2.3. Instruments

**Sociodemographic data**: An electronic questionnaire was designed using Google Forms with six sociodemographic questions: sex, age, university degree, province of the school, educational stage and years of teaching experience.

**Teachers’ perceptions about their preparation for inclusive education**:

- They were asked three dichotomous questions about their initial and ongoing training: (1) Do you think that you were properly prepared through your initial preparation to respond to the diversity of your students’ needs? (2) Has ongoing preparation helped you to respond to the diversity of your students’ needs? (3) Would you be willing to attend courses on inclusive education? These basic questions are intended for further comparison between different educational actors and educational stages [50].
- The Evaluation of Teacher Training for Inclusion Questionnaire, CEFI-R [51], was used. The CEFIR-R consists of 19 items, grouped into four dimensions: (1) Conception of diversity (five items), (2) Methodology (five items), (3) Supports (4 items) and Community participation (5 items). Items from Dimension (1) focus on beliefs of the diversity concept; items from Dimension (2) evaluate aspects related to the design and development of an inclusive curriculum; those from Dimension (3) refer to the teachers’ role with their students; and those included in Dimension (4) allow measuring the participation of educational agents in educational practice. Responses are given on a Likert scale from 1 (strongly disagree) to 4 (strongly agree). Authors reported a Cronbach’s alpha value of 0.79, with each factor above the 0.70 thresholds, which are thus considered good values.

### 2.4. Statistical Analysis

The Statistical Package for Social Sciences (Version 23, IBM SPSS, Chicago, IL, USA) was used. The Kolmogorov-Smirnov test was carried out to determine if the data followed a normal distribution. As this assumption was not met, for both the dichotomous questions (Kolmogorov-Smirnov = 0.484; *p* = 0.000; and Bartlett’s = 36.607; *p* = 0.000) and the CEFI-R items (Kolmogorov-Smirnov = 0.300; *p* = 0.000; and Bartlett’s = 3352.626; *p* = 0.000), non-parametric tests were chosen. Pearson’s Chi-Square test was performed to analyze the differences between the three dichotomous questions according to teachers’ sex and educational stage. The Mann–Whitney U test was used to analyze the differences between the responses to the dichotomous questions and the CEFI-R dimensions. This test was also used to analyze the differences between the dimensions according to the sex of the teachers and the educational stage. The Spearman Rho was performed to explore the association between the CEFI-R dimensions and age. Cronbach’s Alpha was used to calculate the reliability of each of the dimensions from the CEFI-R. Reliability values between 0.60 and 0.70 can be considered acceptable, while values between 0.70 and 0.90 are excellent [52].

## 3. Results

### 3.1. Sample Characterization

The socio-demographic characteristics of the study sample can be examined in Table 1.

### 3.2. The Three Dichotomous Questions

Table 2 displays the frequency of the response distribution to the three dichotomous questions about their perceptions of their initial and ongoing readiness for inclusive education, according to sex and the educational stage, using Pearson’s Chi-Square test. These questions are intended to provide a quick overview of whether PE teachers feel that their initial (Question 1) or ongoing preparation (Question 2) has prepared them to deal with student diversity and whether they are willing to improve their preparation in this respect, which has an attitudinal component (Question 3).

Table 3 shows the distribution of frequencies and differences in each dimension of the CEFI-R according to the responses to the three dichotomous questions. The Mann–Whitney U test was used to analyze the differences. In Dimension (1), Conception of Diversity, statistically significant differences were found according to the answers to the three questions. In Dimensions (2), Methodology, and (3), Supports, statistically significant differences were found according to the answers to Questions 2 and 3. Finally, in Dimension (4), Community Participation, statistically significant differences were found according to the answers to Questions 1 and 3.

### 3.3. The CEFI-R Questionnaire

The CEFI-R scores in their four dimensions (Conception of Diversity, Methodology, Supports, and Community Participation) are shown in Table 4, obtained from the median (Me) value of each item in every dimension. Concerning Dimension (1), although men obtained slightly higher values than women, the difference was not statistically significant. Significant difference occurred between the PE teachers working in primary and secondary schools. In Dimension (2), although men obtained higher values than women, and primary school teachers higher than secondary school teachers, the difference was not statistically significant. In Dimension (3), although women obtained slightly higher values than men, and primary school teachers higher than secondary school teachers, these differences were not statistically significant. In Dimension (4), the scores were identical for both sexes and stages, therefore no statistically significant differences were found.

## 4. Discussion

This study aimed to investigate the perception of Spanish PE teachers on their preparation to cope with the variety of educational needs that their students may present. The main findings showed that a significant number of PE teachers reported not feeling prepared to cope with diversity but showing a positive attitude to address the challenges to promote inclusive education, at least as far as their preparation is concerned. Therefore, the hypothesis of this study was confirmed, suggesting that measures to support educational inclusion in the PE lessons could be considered as a valid tool to achieve inclusive and transformative education.

Thus, teachers answered three dichotomic questions about their initial and ongoing preparation. Regarding the first question, 76.5% denied being prepared to deal with the diversity of their students’ educational needs. In this way, one study performed with PE teachers [53] showed that initial preparation was insufficient to develop the competencies related to inclusive education. Concerning the responses to the second question, 71.5% claimed to have been prepared through ongoing preparation to face diversity. However, according to the third question, 88.8% answered that should attend preparation courses related to inclusion. All this suggests that, although teachers express that they have received preparation in this area, they do not feel fully prepared to face this task. Navarro [54] reported that 19% of secondary PE teachers needed more practical experiences, and they consider it important to continue with their preparation to improve their skills to address diversity. These results are in line with those reported by Rojo-Ramos [7] stressing the importance of improved practical experiences during their initial preparation, as well as providing them with the tools to be offered a proper educational response. Ríos-Hernández [55] indicate that perhaps there is a lack of emphasis on inclusive education.

The studies reviewed indicate some PE teachers have negative attitudes towards inclusive PE lessons [56], but half of them express the need for proper preparation and resources to develop their competencies to teach students with special educational needs [55]. They are aware that PE lessons, due to their organization and idiosyncrasy, offer an opportunity for participation and the promotion of coexistence of all students [57]; however, they are more negative to the idea of including students with emotional-behavioral, attention and learning problems, than those with other difficulties [45], believing that the organization and management of these students are highly demanding [58]. Thus, smaller class sizes would positively affect teachers’ attitudes and self-efficacy [59]. Attitudes and perceptions towards inclusion are extremely complex and vary from country to country and from school to school [60]. Previous findings suggest a strong association between teacher attitudes towards inclusion and teacher self-efficacy [38], considering that adapting PE lessons for children with disabilities is complex and requires a lot of experience [41]. Teachers express the need for more specific preparation about how to teach students with severe disabilities, emotional disturbance, hyperactivity and attention deficit [61]. For this reason, teachers’ initial preparation and positive experiences are essential for teachers to perceive themselves as competent [62], highlighting the importance of being culturally responsive and acquiring the knowledge to carry out socially just practices in educational settings [63]. Therefore, teacher education colleges should make a profound reflection to reflect deeply and restructure curricula to emphasise the elements that can make teachers feel motivated and competent for the task of achieving inclusive PE lessons [64]. Furthermore, scientific literature shows a clear need to validate specific tools to measure these aspects depending on the context of each country [65].

The CEFI-R was also used to check sex and educational stage differences. The results of this research reinforce the studies by several authors [7,66,67] stating that teachers are open to inclusive education and are aware of the potential of PE lessons for all the students. In Dimension (2), men showed a slightly higher score than women teachers. Furthermore, in Dimensions (1), Conception of diversity, and (2), Methodology, higher scores were obtained for primary education teachers. In line with this, Cardona [68] reported that early childhood and primary teachers used more inclusive strategies than those from secondary. Authors such as García- García [69] or Wigfield [70] pointed out that in secondary education differences were greater when compared to primary. This may be due to students having different educational preparation in different subjects, to the fact that they are beginning to undergo personal changes typical of their age, to the interaction with the cultural environment, etc. This educational mismatch may increase the risk of decreasing the interest and motivation of students with educational difficulties, leading to frustration of teachers. In this way, Arnáiz [13] showed that teachers at the early childhood education stage have higher levels of satisfaction with the inclusive process than teachers at the primary education stage and above. Moreover, again Arnáiz [13] stated that teachers sometimes find it difficult to adapt their classes to the group characteristics. Even so, most of the time, PE teachers perceive themselves as having enough didactic and curricular competence to adapt to their students [57]. In Dimension (3), Supports, values were similar both in men and women and in primary and secondary educational stages. The highest score was for item 11 “Joint teacher-support teacher planning would make it easier for support to be provided within the classroom.” This agrees with Arnáiz [13] and Ballús [71] who highlighted the benefits of including a support teacher providing a feeling of security to regular teachers. Ríos-Hernández [55] emphasized the importance of receiving support from teachers who are experts in this subject, such as special education teachers, given the lack of preparation often perceived among teachers. However, del Cueto [57] considered that with good preparation, resources and materials, as well as coordination with other professionals, there would be no need for supporting staff. In Dimension (4), Community Participation, the participants showed high values on the need for the participation of all the agents involved in education, working in synergy with families, and considering the context and resources of the school’s immediate surroundings. Other authors agree with this, as the involvement of all agents in the educational process is essential [72]. Thus, Arnáiz et al. [13] found that in schools where better attitudes towards inclusion were promoted among the whole community, better results were obtained in all areas of learning. Although the results obtained in the CEFI-R four dimensions were quite positive, it is important to highlight that statistically significant differences were only found in Dimension (1), Conception of diversity.

This research has several limitations. The sample size was limited. No questions were included about the specific courses that participants took during their initial or ongoing education, so it is not possible to predict which type of courses enhance readiness for inclusion. For future studies, it can be proposed to extend this study to other regions and territories, as well as to involve other educational stages such as early childhood education and teachers of other levels of education. Another future aim would be to carry out a more in-depth analysis based on the descriptive data of the CEFI-R dimensions according to the dichotomous questions about teachers’ initial and ongoing preparation (Table 3), considering the results obtained.

## 5. Conclusions

PE teachers believe that they have not received the necessary initial preparation and that they consider it important in order to assist in ongoing courses and to deal effectively with the diversity of their students. They are aware of the importance of inclusive education, showing that they should carry out more hours of practice with these students for proper teaching. They perceive greater difficulties at the secondary stage. The participation and collaboration of the whole of society, the educational environment and educational agents should be as coordinated as possible.

PE teachers showed a good predisposition to teach students with specific educational support needs; statistically significant differences were found in the teachers’ educational stage through CEFI-R Dimension (1).

## Figures and Tables

**Table 1:** Sample characterization (*n* = 260).

Variable	Categories	*n*	%
Sex	Men	140	53.8
	Women	120	46.2
Age	Under 30	26	10
	Between 30 and 40	84	32.3
	Between 41 and 50	91	35
	Over 50	59	22.7
University Degree	Primary Education	121	46.54
	Sport Sciences	100	38.46
	Both degrees	39	15
Province of the school	Cáceres	93	35.8
	Badajoz	167	64.2
Educational Stage	Primary	142	54.6
	Secondary	118	45.4

*n*: number; %: percentage.

**Table 2:** Distribution of the three dichotomous questions and responses according to sex and educational stage.

			Yes	No	*p*
**(Question 1) Do you think that you were properly prepared through your initial preparation to respond to the diversity of your students’ needs?**					
Sex	Men	*n* (%)	33 (23.6)	107 (76.4)	0.96
	Women	*n* (%)	28 (23.3)	92 (76.7)	
	Total	*n* (%)	61 (23.5)	199 (76.5)	
Educational Stage	Primary	*n* (%)	35 (24.6)	107 (75.4)	0.62
	Secondary	*n* (%)	26 (22)	92 (78)	
	Total	*n* (%)	61 (23.5)	199 (76.5)	
**(Question 2) Has ongoing preparation helped you to respond to the diversity of your students’ needs** **?**					
Sex	Men	*n* (%)	104 (74.3)	36 (25.7)	0.28
	Women	*n* (%)	82 (68.3)	38 (31.7)	
	Total	*n* (%)	186 (71.5)	74 (28.5)	
Educational Stage	Primary	*n* (%)	105 (73.9)	37 (26.1)	0.34
	Secondary	*n* (%)	81 (68.6)	37 (31.4)	
	Total	*n* (%)	186 (71.5)	74 (28.5)	
**(Question 3) Would you be willing to attend courses on inclusive education?**					
Sex	Men	*n* (%)	126 (90)	14 (10)	0.52
	Women	*n* (%)	105 (87.5)	15 (12.5)	
	Total	*n* (%)	231 (88.8)	29 (11.2)	
Educational Stage	Primary	*n* (%)	133 (93.7)	9 (6.3)	<0.01
	Secondary	*n* (%)	98 (83.1)	20 (16.9)	
	Total	*n* (%)	231 (88.8)	29 (11.2)	

Significant *p*-values are shown in bold. *p* of the Pearson’s Chi-Square test.

**Table 3:** Analysis of the differences of each dimension of the CEFI-R according to the answers to the dichotomous questions.

Dimensions	Question 1			Question 2			Question 3		
	Yes	No	*p*	Yes	No	*p*	Yes	No	*p*
1. Conception of Diversity	3.4 (1.1)	3 (1.2)	<0.01	3.2 (0.8)	2.8 (1.2)	<0.01	3.2 (0.8)	2.8 (1.2)	<0.01
2. Methodology	3 (0.9)	3 (0.8)	0.34	3 (0.8)	2.6 (1)	<0.01	3 (0.8)	2.6 (1)	0.01
3. Supports	2.2 (0.6)	2.4 (0.8)	0.08	2.4 (0.8)	2.2 (0.8)	0.01	2.4 (0.8)	2.2 (0.8)	<0.01
4. Community Participation	3.6 (1)	3.8 (0.8)	0.04	3.6 (0.8)	3.8 (0.8)	0.13	3.6 (0.8)	3.8 (0.8)	<0.01

Each score obtained is based on a Likert scale (1–4): 1 being “Strongly Disagree”, 2 “Partially Disagree”, 3 “Partially Agree” and 4 “Strongly Agree”.

**Table 4:** CEFI-R descriptive analysis and differences of each Dimension, searching for differences between sex and educational stage.

Dimensions	Total	Sex		*p*	Educational Stage		*p*
	Me (IQR)	Men	Women		Primary	Secondary	
1. Conception of Diversity	3 (1)	3.2 (1.2)	3 (1)	0.51	3.2 (1.05)	3 (1.2)	0.02
2. Methodology	3 (0.8)	3 (0.8)	2.8 (0.8)	0.38	3 (1)	2.8 (0.8)	0.34
3. Supports	3 (0.75)	3 (0.75)	3 (1)	0.10	3 (1)	3 (0.75)	0.44
4. Community Participation	3.8 (0.8)	3.8 (0.8)	3.8 (0.8)	0.33	3.8 (0.8)	3.8 (0.8)	0.82

Me = median value; IQR = Interquartile Range. Each score obtained is based on a Likert scale (1–4): 1 being “Strongly Disagree”, 2 “Partially Disagree”, 3 “Partially Agree” and 4 “Strongly Agree”. The correlation coefficient between the dimensions and the different age ranges was performed using the Spearman test (Table 5). Dimension (1), Conception of diversity, and (4), Community Participation, were found to be significant.

**Table 5:** Correlations between the dimensions and the age group variable.

Dimensions	Age *ρ (p)*
1. Conception of diversity	−0.14 (0.02)
2. Methodology	0.13 (0.83)
3. Supports	−0.71 (0.25)
4. Community Participation	0.95 (<0.01)

Each score obtained on the dimensions is based on a Likert scale (1–4): 1 being “Strongly Disagree”, 2 “Partially Disagree”, 3 “Partially Agree” and 4 “Strongly Agree”. Finally, the reliability CEFI-R dimensions values were: Dimension (1) = 0.80; Dimension (2) = 0.91; Dimension (3) = 0.76 and Dimension (4) = 0.93. In this way, values in Dimensions (1) and (3) are considered satisfactory and the ones in Dimensions (2) and (4) excellent [22].

## Data Availability

The datasets used during the current study are available from the corresponding author on reasonable request.
